# The αvβ6 integrin specific virotherapy, Ad5_NULL_-A20.FCU1, selectively delivers potent “in-tumour” chemotherapy to pancreatic ductal adenocarcinoma

**DOI:** 10.1038/s41416-024-02869-3

**Published:** 2024-10-05

**Authors:** Luned M. Badder, James A. Davies, Valerie S. Meniel, Mahulena Marušková, Beatriz Salvador-Barbero, Rebecca J. Bayliss, Toby J. Phesse, Catherine Hogan, Alan L. Parker

**Affiliations:** 1 Division of Cancer and Genetics, Cardiff University School of Medicine, Heath Park, Cardiff, CF14 4XN UK; 2 European Cancer Stem Cell Research Institute, School of Biosciences, Cardiff University, Hadyn Ellis Building, Cardiff, CF24 4HQ UK; 3 The Peter Doherty Institute for Infection and Immunity, The University of Melbourne, Melbourne, VIC Australia; 4 Systems Immunity University Research Institute, Cardiff University School of Medicine, Heath Park, Cardiff, CF14 4XN UK

**Keywords:** Pancreatic cancer, Targeted therapies, Chemotherapy, Adenovirus

## Abstract

**Background:**

Pancreatic ductal adenocarcinoma (PDAC) represent an unmet clinical need. Approximately 90% of PDACs express high levels of αvβ6 integrin. We have previously described Ad5_NULL_-A20, an adenovirus vector with ablated native means of cell entry and retargeted to αvβ6 integrin by incorporation of an A20 peptide.

**Methods:**

Here, we incorporate suicide genes FCY1 and FCU1 encoding for cytosine deaminase (CDase) or a combination of CDase and UPRTase, capable of catalysing a non-toxic prodrug, 5-FC into the chemotherapeutic 5-FU and downstream metabolites, into replication-deficient Ad5 and Ad5_NULL_-A20.

**Results:**

We show that Ad5_NULL_-A20 enables the transfer of suicide genes to αvβ6 integrin-positive PDAC cells which, in combination with 5-FC, results in cell death in vitro which is further mediated by a bystander effect in non-transduced cells. Intratumoural delivery of Ad5_NULL_-A20.FCU1 in combination with intraperitoneal delivery of 5-FC further results in tumour growth inhibition in a cell line xenograft in vivo. Using clinically-relevant 3D organoid models, we show selective transduction and therapeutic efficacy of FCU1 transgenes in combination with 5-FC.

**Conclusion:**

Taken together these data provide the preclinical rationale for combined Ad5_NULL_-A20.FCU1 plus 5-FC as a promising targeted therapy to mediate “in-tumour chemotherapy” and merits further investigation for the treatment of PDAC patients.

## Background

Pancreatic ductal adenocarcinoma (PDAC) is the most common form of human pancreatic cancer. It frequently presents at an advanced stage and has an extremely poor prognosis, with a 5 year overall survival rate of less than 9% [1]. Effective treatment options for PDAC are limited due to the typically late presentation of disease and intrinsic biology which renders it highly resistant to treatment. PDAC patients rely heavily on surgical resection and chemotherapy, which are most effective only in early stages of disease [2]. The lack of validated targeted therapies is limiting for treatment and there remains an urgent unmet clinical need for the development of more effective targeted therapeutics.

Oncolytic virotherapies represent a promising strategy for the treatment of cancer due to their ability to infect, spread and induce immune-stimulatory lytic effects in tumour cells [3, 4]. Adenovirus (Ad)-based oncolytic therapies are particularly well-studied and have been previously adapted to express therapeutic transgenes to enhance tumour cell killing or elicit an anti-tumour host immune response [5–7]. Despite this, the development of Ad5-based cancer-targeting therapies into the clinic has been somewhat hampered due to the limited ability of Ad5 to selectively target tumours following systemic delivery, resulting in poor efficacy. Each major capsid protein of Ad5 play a key role in sequestration by multiple tissue types, including the liver [8], spleen and lung [9], limiting the application of this platform as a cancer-targeting agent. The binding of Ad5 to host cells via coxsackie and adenovirus receptor (CAR) is mediated by the fiber knob protein [10], and is subsequently internalised via αvβ3/5 integrins binding of the penton base protein RGD motifs [11]. CAR is ubiquitously expressed throughout multiple cell types in the body, including erythrocytes, and within tight junctions [12]. Transduction of Ad5, in particular when in contact with blood, can also be mediated by the hexon protein binding to human coagulation factor 10 (FX), resulting in uptake into hepatocytes via cellular heparan sulphate proteoglycans (HSPGs) [13], promoting off-target sequestration in the liver with potential dose limiting toxicities and poor clinical efficacy. Importantly, these dose limiting interactions also deplete the pool of vector available for “on target” activity against tumour cells.

Previously, we developed an Ad5-based vector, Ad5_NULL_-A20, containing tropism modifications to prevent interactions associated with native cellular infection and further engineered to provide selectivity towards tumour cells [14, 15]. Each of the Ad5 major capsid proteins were modified to ablate CAR binding (through modification of the fiber protein), FX interactions (through mutation of the hexon HVR regions), and αvβ3/5 integrin binding (through modification of the penton base). The resulting basal vector was then engineered to incorporate a 20 amino-acid peptide A20 (NAVPNLRGDLQVLAQKVART) within the viral fiber-knob HI loop, capable of binding to αvβ6 integrin with high affinity to facilitate tumour selectivity by a surrogate means of cell entry. αvβ6 integrin overexpression is linked to many solid tumours and is a particularly relevant target for PDAC whereby approximately 90% of patient tumours exhibit high levels of αvβ6, whilst absent or undetectable in the normal surrounding tissue [16, 17].

In this study, we sought to enhance the tumour-selective cytotoxicity of our Ad5_NULL_-A20 platform by incorporation of yeast-derived suicide genes, to develop a virus-directed enzyme prodrug therapy (VDEPT). We engineered replication deficient Ad5 and Ad5_NULL_-A20-based vectors to overexpress either cytosine deaminase (CD; FCY1) or a bifunctional chimeric protein (FCU1) that combines the enzymatic activities of CD and uracil phosphoribosyltransferase (UPRTase). When combined with a relatively non-toxic prodrug, 5-fluorocytosine (5-FC), CD catalyses the direct conversion of 5-FC into the toxic metabolites 5-fluorouracil (5-FU), whilst UPRTase catalyses 5-FC to 5-fluorouridine monophosphate (5-FUMP), bypassing potential mechanisms for therapeutic resistance to 5-FU by direct inhibition of thymidylate synthase (TS) [18]. We found that we were able to selectively transduce αvβ6 integrin+ pancreatic cell lines with Ad5_NULL_-A20.FCY1 and FCU1, whilst demonstrating that CAR+ cells were susceptible to Ad5.FCY1 and Ad5.FCU1 transduction. The transduction of pancreatic cancer cell lines with FCY1- and FCU1- Ads resulted in sensitivity to 5-FC. We demonstrate improved therapeutic efficacy when 5-FC was administered to Ad5_NULL_-A20.FCU1- transduced cells compared to Ad5_NULL_-A20.FCY1. We further demonstrated potential therapeutic impact by testing our viruses using clinically relevant 3D organoid models. We showed successful therapeutic efficacy in organoids derived from both the KPC (*Pdx1-Cre*^ERT^ *LSL-Kras*^G12D/+^*; LSL-Trp53*^*R172H*^*; Rosa26*^LSL-tdRFP^) mouse, a gold standard genetically engineered mouse model (GEMM) of PDAC, as well as from PDAC patients. We further show effective antitumour effect of Ad5_NULL_-A20.FCU1 in combination with 5-FC in vivo using a CFPAC1 subcutaneous xenograft model. Overall, our results suggest a novel application for a selective and potent candidate for “in tumour chemotherapy” using Ad5_NULL_-A20 in the clinical setting.

## Materials and methods

### Viral vector generation

Adenovirus vectors were generated using genetic modifications by AdZ homologous recombineering using previously described methods [14]. Replication-deficient vectors based on a wild type Ad5 genome were modified to generate the Ad5_NULL_-A20 platform. Ablation of CAR binding was achieved via the KO1 mutation in the AB loop of the L5 fiber knob gene; ablation of binding to coagulation factor 10 via a mutation in hypervariable region 7 of the L3 hexon gene; ablation of αvβ3/5 integrin binding via RGD-RGE mutation in the L2 penton base gene. Retargeting of the modified Ad genome to αvβ6 integrin was achieved by insertion of an A20 peptide sequence from FMDV (NAVPNLRGDLQVKVART) into the viral fiber knob HI loop (between residues G546 and D547). Gene synthesised FCY1 and FCU1 (codon optimised) PCR fragments were inserted under the control of a CMV promoter replacing a sacB cassette selectable marker.

Viruses were produced in either T-REx-293 (Ad5) or HEK293-β6 (β6-expressing) cell lines. DNA was amplified using a maxiprep kit as per the manufacturer’s guidelines. Virus particles were generated by transfection in a T25 CellBIND flask (Corning) of T-REx-293 (Ad5) or HEK293-β6 cells (β6-expressing) cells. When cytopathic effect (CPE) was observed in cells, cells were collected, and virus was further amplified in expanded cells (10 X T150 CellBIND flasks). Caesium chloride (CsCl) two-step purification method was used to extract purified viruses. Viral particles/mL (vp/mL) were quantified by micro-BCA assay (Thermo Fisher, Loughborough, UK) using the equation 1 µg protein = 4 × 10^9^ viral particles (vp)

### Cell lines and culture

Human pancreatic cancer cells, CFPAC1, MIA PaCa-2, PT45, (American Type Culture Collection (ATCC)) were maintained in Dulbecco’s modified Eagle’s medium (DMEM, Sigma-Aldrich, New York, NY, USA) supplemented with 10% heat-inactivated foetal bovine serum (FBS, Gibco, Grand Island, NY, USA), 1% penicillin/streptomycin (P/S, Gibco, Paisley, UK), 1% L-glutamine (Gibco). ASPC1, BxPC3 and PANC10.05 (ATCC) cells were maintained in Roswell Park Memorial Institute (RPMI) 1640 Medium (Sigma) supplemented with 10% FBS, 1% P/S, 1% L-glutamine. All cultures were maintained in a 5% CO2 humidified atmosphere at 37 °C. All cell lines were routinely tested for mycoplasma using MycoAlert Mycoplasma detection kit (Lonza).

### Cell viability assay

Cells were seeded at a density of 5000 cells per well in triplicate in white clear-bottomed 96 well plates. Following 24 h in culture, cells were transduced with indicated viral vectors, within a range of 500–5000 viral particles (vp) per cell. Viruses were diluted in serum-free growth medium and cells were transduced for 24 h at 37 °C. Cells were then washed with PBS and incubated in full growth medium containing a dilution of 5-FC (Sigma-Aldrich, Gillingham, UK) or PBS vehicle control for 3 days. Cell viability was measured using CellTiter-Glo® Luminescent Cell Viability Assay (Promega, Madison, WI, USA) as per the manufacturer’s instructions, and luminescence was measured with a multimode plate reader (Bio-Rad, Hertfordshire, UK). Relative luminescence units were normalised to the vehicle control, with FCU1- and FCY1- viruses normalised to parental virus controls.

### Mouse pancreatic organoids

Pdx-1 Cre^ERT^ [19], LSL-Kras^G12D/+^ [20]; LSL-Trp53^R172H/+^ [21]; Rosa26^LSL-tdRFP^ [22] (KPC) mouse lines have all been previously described. KPC lines were used to generate pancreatic tumour-derived organoids as described previously [23, 24]. Animals were housed in conventional pathogen-free animal facilities and all procedures were conducted in accordance with the UK Home Office regulations (ASPA 1986 & EU Directive 2010) under Home Office approved Project licence granted to CH and under the guidelines of Cardiff University Animal Welfare and Ethics Committee. Mice were genotyped by PCR analysis using sequences as described in [24]. Cre recombinase was induced by administering a single intraperitoneal injection of tamoxifen (1 µg/40 g) to 6–8-week-old mice [24]. Typically, KPC mice develop PDAC tumours at 20–22 weeks post induction of Cre recombinase [25]. Tumour-bearing pancreas was harvested and routinely dissected for downstream analyses or organoid culture. For generation of organoids, mouse pancreas was mechanically dissociated before digestion in collagenase Type 1 (Sigma-Aldrich) and Dispase II (Sigma-Aldrich) to a concentration of 0.125 mg/mL at 37 °C. Following several washes in HBSS supplemented with 5% FBS, tissue was passed through a 40 μm cell strainer. The washed cells were then resuspended in Matrigel (Corning, Bedford, MA, USA) and seeded within individual domes in 24 well plates. Once polymerised, pancreatic cells were overlaid with expansion medium containing Advanced DMEM F12 (Gibco, Grand Island, NY, USA) supplemented with HEPES (1%, Gibco, Paisley, UK), GlutaMAX (1% Gibco, Paisley, UK)), 1% penicillin/streptomycin(P/S, Gibco), B27 (1 X ThermoFisher Scientific, MA, USA), N2 (1 X, ThermoFisher Scientific), 1.25 mM n-acetyl-L-cysteine (Sigma-Aldrich, MO, USA), R-spondin 1 conditioned medium (5%) or recombinant R-spondin (1 µg/ml; Peprotech, CA, USA), 10 mM Nicotinamide (Sigma-Aldrich), 10 nM recombinant human [Leu15] Gastrin I (Sigma-Aldrich), 50 ng/mL recombinant EGF (Peprotech, CA, USA), 100 ng/mL recombinant human FGF10 (Peprotech, NJ, USA) and 25 ng/mL recombinant human Noggin (Peprotech, NJ, USA) and incubated under standard tissue culture conditions (37 °C, 5% CO2).

### Immunohistochemical staining

Tumour sections from tissues were mounted on slides prior to serial washes in xylene and graded ethanol (100%, 90% and 70%). Protease antigen retrieval was carried out for αvβ6 integrin by adding protease 2 (0.1 mg/ mL, Roche, Basel, Switzerland) to sections at 37 °C for 12 min. Slides were washed and incubated with 1% H_2_O_2_ for 15 min at room temperature, washed, and blocked using 2.5% horse serum for 30 min at room temperature. αvβ6 integrin primary antibody was added to slides (1:750; EM05201, Absolute Antibody) in 1% BSA/PBS at 4 °C overnight. Primary antibody was removed and replaced with diluted ImmPACT DAB chromogen (Vector Labs, Newark, CA, USA) for 4 min at room temperature. Slides were submerged in Mayer’s haematoxylin prior to rinsing in ddH_2_O. Slides were dehydrated prior to mounting with DPX mountant before imaging.

### Organoid viability assay

Organoids established in culture were mechanically disaggregated into fragments, with a representative population further digested to single cells by incubation in TrypLE™ Express (Gibco, Grand Island, NY, USA) at 37 °C for counting purposes. Single cell counts were carried out using an automated cell counter (Cell Drop, DeNovix) and used to estimate cell numbers within organoid fragments. Organoid fragments were incubated with viruses at a dose of 5000 vp/cell for 30 min at 37 °C. Following incubation, tubes were transferred to ice and the medium containing transduced organoids (10% final volume) was supplemented with Matrigel (90% final volume), mixed and seeded in 5–10 µL drops in triplicate in white clear-bottomed 96 well plates. Plates were incubated at room temperature for 5 min, prior to inverting plates and incubating for 1 one hour at 37 °C to enable Matrigel polymerisation. Growth medium containing 10 µM ROCK inhibitor, Y-27632, (BD Biosciences, CA, USA) was overlaid on Matrigel domes and incubated under standard tissue culture conditions for 24 h. Following a 24 h incubation, medium was removed from wells and replaced with growth medium containing a dilution of 5-FC or PBS vehicle control for a further 5–6 days as required. Cell viability was measured as indicated after drug treatment using Cell Titer Glo® (Promega) assay. Cell Titer Glo® reagent was added to a total volume of 50 µL per well and placed on a shaker platform (595 rpm) for 1 h at room temperature, protected from light. Luminescence was measured on a multimodal platereader (BioRad) and values expressed as a percentage viability relative to vehicle control cells.

### Cell surface receptor staining

To assess cell surface receptors by flow cytometry, cells were harvested, washed in 5% FBS/PBS and added at a density of 100,000 cells per well in a v-bottomed 96 well plate (Nunc) and incubated on ice for 1 h with the respective primary mouse mAb; Anti-CAR (RmcB, 3022487; Millipore, Watford, UK) and anti-αvβ6 (10D5, MAB2077Z; Millipore) and matched IgG control were used at a concentration of 1:500 or 1:1000 for a matched IgG control. Cells were then washed and incubated on ice for 30 min with 1:1000 dilution of Alexa-647 labelled goat anti-mouse F(ab’)2 (A-21237; Life Technologies, Paisley, UK) or Alexa-488 labelled goat anti-mouse F(ab’)2. Stained cells were fixed using 4% paraformaldehyde prior to measurement by flow cytometry on Accuri C6 (BD Biosciences). For flow cytometry, a minimum of 10,000 events were acquired. Analysis was performed using FlowJo v.10 (FlowJo, LLC) by sequential gating on cell population, singlets and Alexa-647 or Alexa-488 positive cells.

### Patient derived organoids

Patient-derived organoids (PDOs) from pancreatic tumours HCM-CSHL-0079-C25 (ATCC® PDM30™); HCM-CSHL-0089-C25 (ATCC® PDM36™) HCM-CSHL-0091-C25 (ATCC® PDM38™); HCM-CSHL-0092-C25 (ATCC® PDM39™) were acquired from the ATCC repository and cultured according to manufacturer’s instructions. We used models and data derived by the Human Cancer Models Initiative (HCMI) https://ocg.cancer.gov/programs/HCMI; dbGaP accession number phs001486. PDOs were cultured in Matrigel (100% v/v, Corning, Beford, MA, USA) and maintained at a seeding density of 0.25–1 × 10^6^ cells/ 100 µL of Matrigel per well of a 6-well plate. A complete medium change was carried out every 3-4 days in culture. Organoids were maintained in Advanced DMEM F12 (Gibco, Grand Island, NY, USA) supplemented with HEPES (1%, Gibco, Paisley, UK), GlutaMAX (1% Gibco, Paisley, UK)), 1% penicillin/streptomycin (P/S, Gibco), 1X B-27 (ThermoFisher Scientific, MA, USA), Wnt3A Conditioned medium (50%), R-spondin Conditioned medium (10%), 1.25 mM n-acetyl-L-cysteine (Sigma-Aldrich), 10 mM Nicotinamide (Sigma-Aldrich), 10 nM recombinant human [Leu15] Gastrin I (Sigma-Aldrich), 50 ng/mL recombinant EGF (Peprotech, CA, USA), 100 ng/mL recombinant human FGF10 (Peprotech, NJ, USA), 100 ng/mL recombinant human Noggin (Peprotech, NJ, USA) and 500 nM A 83-01 (Peprotech) and incubated under standard tissue culture conditions (37 °C, 5% CO2). Organoids were split by enzymatic digestion when the appropriate confluence was reached, using TrypLE Express dissociating agent. 10 µM ROCK inhibitor (BD) was added to growth medium for the first 3 days after splitting.

### In vivo efficacy studies

Athymic nude mice were purchased from Charles River (UK) and allowed to acclimatise for a minimum of 7 days prior to experiments. All procedures were conducted in accordance with the UK Home Office regulations (ASPA 1986 & EU Directive 2010) under Home Office approved Project licence granted to TP and under the guidelines of Cardiff University Animal Welfare and Ethics Committee. Mice were housed in filtered cages. Subcutaneous CFPAC1 models were established by injecting 2 × 10^6^ cells into both flanks. 7 days post engraftment, 3 × 10^10^ vp of Ads or PBS control were injected intratumourally. 24 h post I.T injection, 5-FC (200 mg/kg) or vehicle control (PBS) was administered daily by I.P injection for 8 days, then again at days 22 and 25. Tumours were measured with calipers every 1–3 days to ensure tumour volumes did not exceed 1.5 cm throughout the experiment, and the volume was calculated as *V* (mm^3^) = π/6 × *W*^2^ × *L* and normalized to day 0 post I.T treatment.

### Viral genome copy number analysis of in vivo tumours

DNA was extracted from snap frozen tissues using DNeasy Blood and Tissue DNA extraction kit (Qiagen) according to the manufacturer’s protocol. DNA concentration was determined using a NanoDrop photospectrometer. 25 ng of total DNA was subjected to quantitative PCR using Fast SYBR Green Master Mix. Reactions were performed in technical triplicate, using primers for the hexon region of the genome. Standard curves were prepared by serial dilution of replication-deficient Ad5.

### Statistical analysis

Statistical analyses were performed using GraphPad (San Diego, CA) Prism software. Data are presented as mean ± standard error of the mean unless otherwise specified. Experiments were performed to *n* = 3 independent experiments, unless otherwise stated. Statistical analysis was carried out as indicated and statistical significance is shown as follows; ns = *p* > 0.05; *p < 0.05; **p < 0.01; ***p < 0.001; ****p < 0.0001.

Graphical depictions were created using BioRender.com

## Results

### Replication-deficient Ad5 expressing FCY1/FCU1 transduce CAR+ pancreatic cells and sensitise cells to 5-FC treatment in vitro

**Fig. 1:**
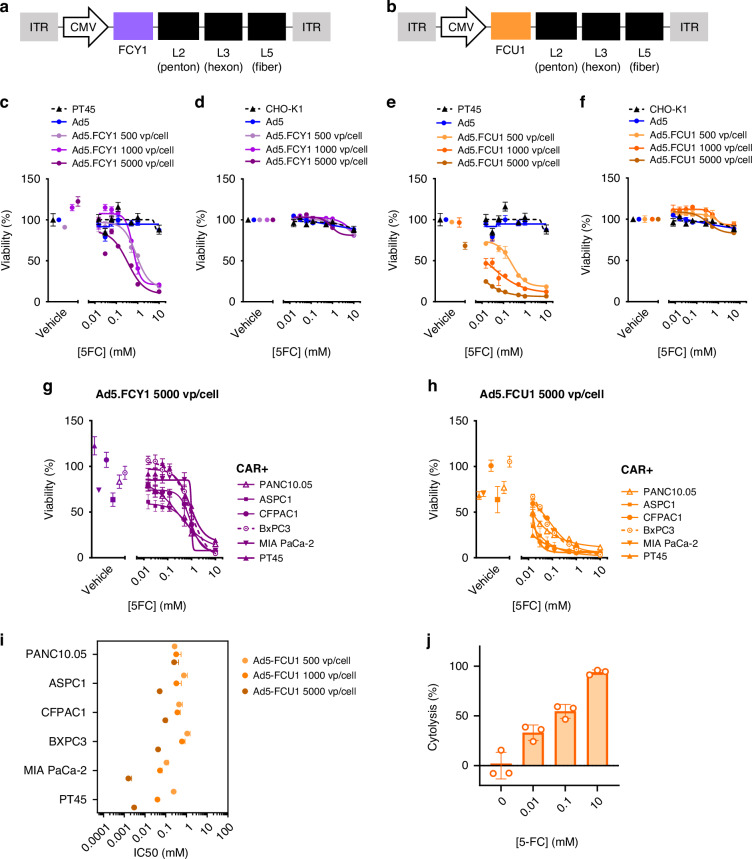
Replication-deficient Ad5 encoding cytosine deaminase and UPRTase sensitises CAR+ pancreatic cells to 5-FC. **a** Schematic representation of Ad5 expressing the FCY1 gene. **b** Schematic representation of Ad5 expressing the FCU1 gene. **c**, **d** Ad5.FCY1 sensitises CAR+ cells to 5-FC. Dose-response survival curves of PT45 (CAR+) (**c**) and CHO-K1 (CAR-) (**d**) cells exposed to 5-FC for 3 days following transduction with 500, 1000 and 5000 vp/cell of Ad5.FCY1. Cell viability was estimated by Cell-Titer Glo®. Data are represented as mean ± SEM from *n* = 3 technical replicates. **e**, **f** Ad5.FCU1 sensitises CAR+ cells to 5-FC. Dose-response survival curves of PT45 (**e**) and CHO-K1 (**f**) cells exposed to 5-FC for 3 days following transduction with 500, 1000 and 5000 vp/cell Ad5.FCU1. Cell viability was estimated by Cell-Titer Glo®. Data are represented as Data are represented as mean ± SEM from *n* = 3 technical replicates. **g**, **h** Dose-response survival curves of multiple pancreatic cell lines treated with 5-FC following transduction with 5000 vp/cell Ad5.FCY1 (**g**) and Ad5.FCU1 (**h**) Cell viability was estimated by Cell-Titer Glo®. Data are represented as mean ± SEM, *n* = 3. **i** Pancreatic cell line response (IC_50_) to a dose range of 5-FC following transduction with 500–5000 vp/cell of Ad5.FCU1 quantified by CellTiter Glo®. Cells were treated with 5-FC for 3 days following 24 h transduction with Ad5_NULL_-A20.FCU1. Dots represent IC_50_ estimates from biological repeats. Mean effects are shown and error bars represent S.E.M from *n* = 3 biological repeats. **j** Bar chart illustrating cytolysis (%) in PT45 cells treated with 5-FC. Cells were transduced with Ad5.FCU1 for 24 h prior to treatment with 5-FC and measured for changes in impedance over time. Data are mean ± SD from *n* = 3 technical replicates.

To investigate whether Ad5-mediated expression of cytosine deaminase (FCY1) and a bifunctional chimeric protein encoding cytosine deaminase and UPRTase (FCU1) would catalyse the conversion of 5-FC into toxic substrates in pancreatic cells, we generated two Ad5 vectors bearing either the FCY1 or FCU1 transgene. Using homologous recombination based recombineering, the coding sequence of both FCY1 and FCU1 were successfully introduced into the deleted E1 region of Ad5 under the CMV IE promoter as shown in the schematic of the constructed viruses (Fig. 1a, b). Successful insertion of the FCY1 or FCU1 gene were confirmed by sequencing (data not shown).

To assess whether expression of cytosine deaminase (CD) by Ad5.FCY1 or cytosine deaminase/UPRTase by Ad5.FCU1 would sensitise cells to 5-FC, we transduced cells with known levels of CAR expression [15] with Ad5.FCY1 or Ad5.GFP at 500, 1000 and 5000 viral particles/cell (vp/cell) for 24 h. Cells were then exposed to 5-FC prodrug at varying concentrations for 3 days prior to assessing cell viability using an ATP-based assay readout. Ad5.FCY1-transduced PT45 (CAR^high^) pancreatic cancer cells were susceptible to 5-FC treatment in a dose-dependent manner compared to non-transduced cells, unlike those transduced with Ad5.GFP as an Ad5 based transgene control (Fig. 1c). Conversely, CHO-K1 (CAR^negative^) cells showed no difference in viability in the presence of 5-FC following transduction with Ad5.FCY1 (Fig. 1d). Transduction of PT45 cells with Ad5 expressing FCU1 resulted in a greater sensitisation to 5-FC treatment compared to FCY1, with IC_50_ values of 0.03 mM at the highest virus dose of 5000 vp/cell in Ad5.FCU1, compared with 0.85 mM in Ad5.FCY1 (Fig. 1e), corroborating previous studies in the literature demonstrating the potency of CD and UPRTase in combination with 5-FC compared to CD alone [5, 26]. Conversely, CAR^negative^ CHO-K1 cells showed no difference in viability in the presence of 5-FC when transduced with Ad5.FCY1 or Ad5.FCU1 (Fig. 1f) suggesting that the presence of CAR was essential to mediate Ad5 based 5-FC dependent cytotoxicity, as would be expected given the primary receptor for Ad5 is CAR.

We next evaluated the sensitivities of PANC10.05, BxPC3, CFPAC1, MIA PaCa-2 and ASPC1 pancreatic tumour cells after transduction with Ad5.FCY1, Ad5.FCU1 or Ad5.GFP, alone or in combination with 5-FC. A dose range of 5-FC treatment alone or in combination with Ad5.GFP had no impact on cell viability in all cell lines tested compared with vehicle only control (Supplementary Fig. S1A–D). CAR+ pancreatic cells were sensitised to 5-FC treatment following transduction with Ad5.FCY1 (Fig. 1g, Supplementary Fig. S1A–E) and Ad5.FCU1 (Fig. 1h, Supplementary Fig. S1A–E), with cells transduced with Ad5.FCU1 having the highest sensitivity to 5-FC compared to those transduced with Ad5.FCY1. IC_50_ values of cells transduced with Ad5.FCU1 and treated with 5-FC were lowest in cells with highest CAR expression levels, with PT45 cells exhibiting values of 0.003 mM after transduction with 5000 vp/cell, compared with PANC10.05 with IC_50_ values of 0.25 mM (Fig. 1i). To assess the impact of Ad5.FCU1 in combination with 5-FC over time in culture, we used an impedance-based assay as a readout of overall cell growth. PT45 cells transduced with Ad5.FCU1 and treated with 10 mM 5-FC demonstrated increased cytolysis (93.8%) compared to those treated with virus alone (<1%) (Fig. 1j, Supplementary Fig. S3A, B). Taken together these data confirm that CAR is essential for the entry of Ad5.FCY1/Ad5.FCU1 into cells to mediate prodrug-dependent toxicity, and simultaneous expression of CD and UPRTase can result in a cooperative effect that increases the sensitivity of target cells to 5-FC. Given that CAR is expressed on many organs, tissues and cells [12], and it’s expression is commonly downregulated during tumorigenesis [27], Ad5 based vectors would make a poor candidate for tumour selective VDEPT approaches. Consequently, we sought to evaluate the potential of a highly tumour selective viral platform which exclusively uses αvβ6 integrin for cell entry.

### Replication-deficient Ad5_NULL_-A20 expressing FCY1/FCU1 sensitise αvβ6+ pancreatic cells to 5-FC treatment

**Fig. 2:**
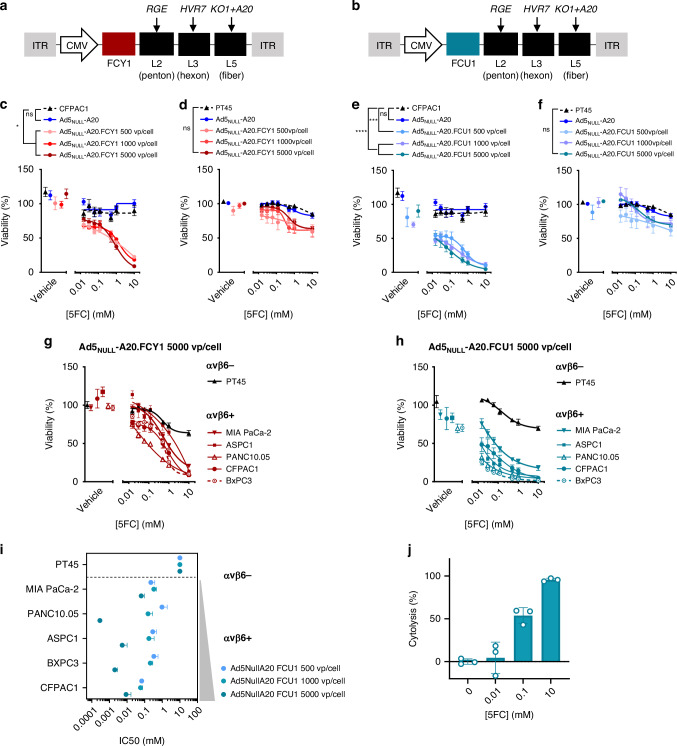
Replication deficient Ad5_NULL_-A20 encoding cytosine deaminase and UPRTase sensitises cells expressing αvβ6 to 5-FC treatment. **a** Schematic representation of Ad5_NULL_-A20 expressing the FCY1 gene. **b** Schematic representation of Ad5_NULL_-A20 expressing the FCU1 gene. **c**, **d** Ad5_NULL_-A20.FCY1 sensitises only αvβ6+ cells to 5-FC. Dose-response survival curves of BxPC3 (αvβ6+) (**c**) and PT45 (αvβ6-) (**d**) cells exposed to 5-FC for 3 days following transduction with 500, 1000 and 5000 vp/cell Ad5_NULL_-A20.FCY1. Cell viability was estimated by CellTiter Glo®. Data are represented as mean ± SEM, *n* = 3. Two-way ANOVA with Tukey’s post hoc test was used to calculate *p* values; *p < 0.05, **p < 0.01, ***p < 0.001, ****p < 0.0001. **e**, **f** Ad5_NULL_-A20.FCU1 sensitises only αvβ6+ cells to 5-FC. Dose-response survival curves of BxPC3 (**e**) and PT45 (**f**) cells exposed to 5-FC for 3 days following transduction with 500, 1000 and 5000 vp/cell Ad5_NULL_-A20.FCU1. Cell viability was estimated by CellTiter Glo®. Data are represented as mean ± SEM, *n* = 3. Two-way ANOVA with Tukey’s post hoc test was used to calculate *p* values; *p < 0.05, **p < 0.01, ***p < 0.001, ****p < 0.0001. Dose-response survival curves of multiple cell lines treated with 5-FC following transduction with 5000 vp/cell Ad5_NULL_-A20.FCY1 (**g**) and Ad5_NULL_-A20.FCU1 (**h**) Cell viability was estimated by CellTiter Glo®. Data are represented as mean ± SEM, *n* = 3. **i** Pancreatic cell line response (IC_50_) to a dose range of 5-FC following transduction with 500–5000 vp/cell of Ad5_NULL_-A20.FCU1 quantified by CellTiter Glo®. Cells were treated with 5-FC for 3 days following 24 h transduction with Ad5_NULL_-A20.FCU1. Dots represent IC_50_ estimates from biological repeats. Mean effects are shown and error bars represent S.E.M from *n* = 3 biological repeats **j**. Bar chart illustrating cytolysis (%) in PT45 cells treated with 5-FC. Cells were transduced with Ad5_NULL_-A20.FCU1 for 24 h prior to treatment with 5-FC and measured for changes in impedance over time. Data are mean ± SD from *n* = 3 technical replicates. Cytolysis was analysed by impedance changes in cells over time.

We previously demonstrated that the elimination of native tropisms of Ad5 (Ad5_NULL_) and incorporation of a 20 amino acid peptide sequence A20 (Ad5_NULL_-A20) can re-target Ad5 towards the tumour cell marker αvβ6 integrin [14], to effectively and selectively transduce pancreatic tumour cells in vitro [15]. To assess whether the cell killing activity of this platform could be enhanced to target αvβ6 integrin+ pancreatic cells, we similarly engineered Ad5_NULL_-A20 to overexpress CD (FCY1) (Fig. 2a) or the bifunctional chimeric protein of CD and UPRTase (FCU1) (Fig. 2b). Successful insertion of the FCY1 or FCU1 gene were confirmed by sequencing (data not shown).

We assessed the impact of cells with previously characterised levels of αvβ6 integrin expression [15] to 5-FC treatment alone or in combination, following transduction with mock, Ad5_NULL_-A20 transgene control, Ad5_NULL_-A20.FCY1 or Ad5_NULL_-A20.FCU1 for 24 h. Cells were treated with 5-FC for 3 days prior to assessing cell viability. CFPAC1 cells (αvβ6^high^) transduced with Ad5_NULL_-A20.FCY1 and subjected to varying doses of 5-FC demonstrated reduced viability compared to mock-transduced cells or those transduced with Ad5_NULL_-A20 transgene control (Fig. 2c). Conversely, PT45 pancreatic cells, previously shown to express limited αvβ6 integrin expression levels [15], showed no significant difference in viability following a combination of Ad5_NULL_-A20.FCY1 and 5-FC compared with mock or transgene control conditions (Fig. 2d). CFPAC1 cells (αvβ6^high^) were further sensitised to 5-FC when transduced with Ad5_NULL_-A20.FCU1 (Fig. 2e), with cell viability impaired at the lowest doses of 5-FC (0.01 mM). High viral titres of Ad5_NULL_-A20.FCU1 failed to sensitise PT45 (αvβ6^low^) cells to 5-FC treatment (Fig. 2f) confirming successful selectivity to αvβ6.

We tested a panel of pancreatic cell lines with known levels of αvβ6 integrin [15] with Ad5_NULL_-A20.FCY1 (Fig. 2g, Supplementary Fig. S2A–D) or Ad5_NULL_-A20.FCU1 (Fig. 2h, Supplementary Fig. S2A–D) in combination with 5-FC and found that cells with the highest levels of αvβ6 integrin were most susceptible to treatment. Ad5_NULL_-A20.FCU1 induced the greatest sensitisation to 5-FC from all vectors tested. Transduction at 5000 vp/cell of Ad5_NULL_-A20.FCU1 led to a dose dependent change in IC_50_ values compared to lowest virus titres (500 vp/cell) in αvβ6 integrin+ cells (Fig. 2i). Overall viability over time as gauged by impedance measurements further showed that Ad5_NULL_-A20.FCU1 transduction in CFPAC1 (αvβ6^high^) cells had no overall impact on cell growth in the first 24 h (Supplementary Fig. S3C). Following 5-FC addition to the media, overall resistance was limited in cells suggesting an impairment to overall growth (Supplementary Fig. S3D). Following 3 days of treatment with 5-FC, there was a significant dose-dependent increase in cytolysis (97.3%) compared to vehicle control conditions (<1%; Fig. 2j). Taken together this suggests that Ad5_NULL_-A20.FCY1 and Ad5_NULL_-A20.FCU1 selectively transduce cells in a αvβ6 integrin dependent manner, resulting in an increased sensitisation to 5-FC in pancreatic tumour cells with high expression levels of αvβ6 integrin.

### FCU1-mediated bystander effects affect overall viability of CFPAC1 pancreatic cancer cells

**Fig. 3:**
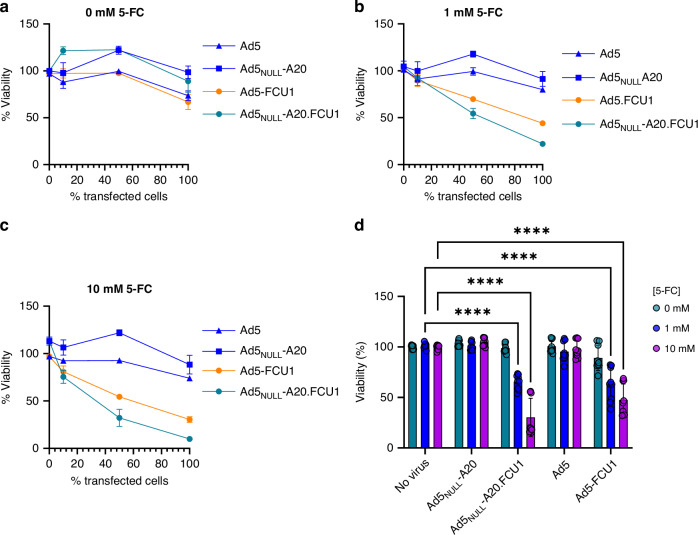
FCU1-mediated bystander effects in CFPAC1 cells. **a**–**c** CFPAC1 cells are sensitised to enzymatic activation of 5-FC by FCU1 transduction of neighbouring cell populations. CFPAC1 cells were transduced with 1000 vp/cell Ad5.FCU1, Ad5_NULL_-A20.FCU1, and respective controls prior to mixing at different ratios with non-transduced cells. Cells were treated with 0 mM (**a**), 1 mM (**b**) and 10 mM (**c**) 5-FC for 3 days, with overall cell viability measured by CellTiter Glo®, and normalised to non-treated control. Error bars indicate S.E.M from *n* = 3 biological replicates. **d** Toxicity of Ad5_NULL_-A20.FCU1 transfection combined with 5-FC impair the viability of untransduced cells. Supernatants were harvested from CFPAC1 cells transduced with 1000 vp/cell of viruses combined with 5-FC and added to naïve CFPAC1 cells. Viability was measured after 3 days in culture using CellTiter Glo®. Error bars indicate the S.E.M from *n* = 3 biological replicates.

We next sought to determine whether FCU1-mediated conversion of 5-FC to 5-FU and 5-FUMP could affect the overall viability of neighbouring untransduced cells. CFPAC1 cells, with relatively similar levels of CAR and αvβ6 integrin expression levels, transduced with Ad5.FCU1 or Ad5_NULL_-A20.FCU1, were mixed with naïve CFPAC1 cells prior to treatment with 5-FC. Overall, no effect on viability was observed in mixed populations of naïve and transduced cells treated with 0 mM 5-FC (Fig. 3a). When naïve cells were mixed at an equal ratio with Ad5_NULL_-A20.FCU1 or Ad5.FCU1-transduced cells and treated with 1 mM (Fig. 3b) and 10 mM doses (Fig. 3c), a sensitisation to 5-FC was observed, even when only 10% of the population was made up of transduced cells. To further investigate the bystander effect of these treatment conditions, we analysed the overall cell viability of CFPAC1 cells following exposure to cell culture supernatant collected from Ad5_NULL_-A20.FCU1-transduced cells treated with 5-FC. Addition of conditioned media at a 1:10 dilution in the absence of 5-FC was shown to have no effect on untransduced cells, in all conditions, including media conditioned from Ad5_NULL_-A20.FCU1 cells (Fig. 3d). CFPAC1 cell viability was significantly reduced when cells were exposed to media conditioned by cells transduced with Ad5.FCU1 and Ad5_NULL_-A20.FCU1 in the presence of 1 mM and 10 mM 5-FC, with a greater cytotoxic effect observed in Ad5_NULL_-A20.FCU1-transduced cells. Taken together this suggests FCU1-mediated conversion of 5-FC to 5-FU is capable of eliciting toxicity to neighbouring naive cells in vitro. This bystander effect could provide a possible advantage by causing cell death in neighbouring tumour cells, independent of viral transduction and expression of CD and UPRTase.

### Ad5_NULL_-A20-mediated FCU1 expression sensitises mouse pancreatic tumour organoids to 5-FC

**Fig. 4:**
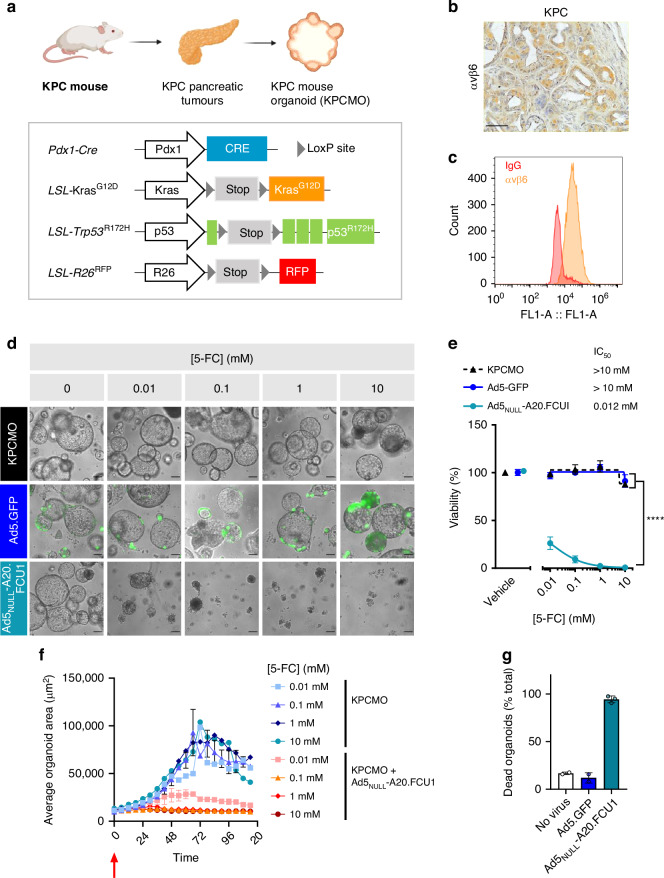
Ad5_NULL_-A20-FCU1 effectively replicates and sensitises KPC-derived mouse tumour organoids to 5-FC treatment. **a** Schematic depicting the KPC mouse model, incorporating Kras^G12D^ point mutation, p53^R172H^ deletion in a Pdx1 pancreatic promoter. **b** Representative αvβ6 integrin immunohistochemistry from KPC mouse model ductal structures showing positive staining. Scale bar = 50 µm. **c** αvβ6 Integrin screening of mouse organoids by flow cytometry. **d** Representative images of KPCMO organoids following 5 days of treatment with 5-FC following mock, Ad5.GFP, and Ad5_NULL_-A20.FCU1 transduction. Scale bar = 100 µm. **e** Ad5_NULL_-A20.FCU1 sensitises mouse pancreatic tumour organoids (KPCMO) to 5-FC treatment. Organoids were transduced with 5000 vp/cell of virus for 24 h prior to treatment with a range of 5-FC doses. Cell viability was estimated by CellTiter Glo®. Data are represented as mean ± SEM, *n* = 3. Two-way ANOVA with Tukey’s post hoc test was used to calculate *p* values; *p < 0.05, **p < 0.01, ***p < 0.001, ****p < 0.0001. **f** Incucyte-generated organoid area plots from KPCMO organoids alone, and KPCMO organoids transduced with Ad5_NULL_-A20.FCU1 treated with 5-FC. Red arrow indicates 5-FC treatment. Data are presented as average organoid area (µm^2^) ± SD from minimum two replicate wells. **g** Relative number of dead organoids quantified from Incucyte images following 4 days of 5-FC (10 mM) treatment. Data are presented as number of dead organoids (% of total) per condition ±SD from minimum two replicate wells.

Given the favourable, tumour-selective targeting observed in vitro, we performed ex vivo studies in organoids derived from the clinically relevant KPC (*Pdx1-Cre*^ERT^ *LSL-Kras*^G12D/+^*; LSL-Trp53*^*R172H*^*; Rosa26*^LSL-tdRFP^) genetically engineered mouse model (GEMM) of PDAC. We administered tamoxifen to induce Pdx1-Cre recombinase to induce tumour formation with KrasG12D and p53R172H transgenes and pancreatic tumours [28]. Following tumour formation, we generated organoid cultures from KPC tumours (Fig. 4a; [23]). To confirm that αvβ6 integrin expression was retained in the KPC mouse model, we evaluated tumour sections by IHC and found that KPC mouse tumours maintained high levels of αvβ6 expression (Fig. 4b). We determined the expression levels of αvβ6 integrin in organoids derived from the KPC mouse (KPCMO) by flow cytometry and found that they expressed high levels of αvβ6 integrin (Fig. 4c).

To assess whether αvβ6 integrin-expressing KPC organoids could be sensitised to 5-FC treatment following transduction with Ad5_NULL_-A20.FCU1 as our candidate therapy, organoids were disaggregated to cell fragments and exposed to 5000 vp/cell of virus for 24 h, then treated with 5-FC for 5 days and assessed for viability. Fluorescence and phase images of KPCMO demonstrate that organoids were amenable to virus transduction, with GFP+ cells observed in organoids transduced with Ad5.GFP as a transgene expressing control (Fig. 4d). We found that transduction of Ad5_NULL_-A20.FCU1 in the absence of prodrug had minimal impact on overall organoid viability as measured by an ATP assay, suggesting that the virus alone was non-toxic to the cells (Fig. 4e). In the presence of 5-FC, a significant effect on overall viability was observed in a dose-dependent manner, with IC_50_ values of 0.012 mM in Ad5_NULL_-A20.FCU1-transduced organoids compared to an IC_50_ of >10 mM in mock and Ad5.GFP transduction conditions (Fig. 4e). We further evaluated the impact of Ad5_NULL_-A20.FCU1 in KPCMO using an Incucyte® assay readout to acquire real-time quantitative data on organoid growth and found that the average organoid area (µm^2^) increased in both mock- and Ad5.GFP transduced organoids, regardless of 5-FC dose (Supplementary Fig. S4A, B). However, the combination of Ad5_NULL_-A20.FCU1 and 5-FC treatment inhibited the growth of KPCMO organoids (Fig. 4f) and resulted in an increase in the number of dead organoids as measured by phenotypic scoring (Fig. 4g, Supplementary Fig. S4C), suggesting the combination of Ad5_NULL_-A20.FCU1 with 5-FC is able to transduce and elicit toxicity in KPCMO organoids. Taken together, our data suggest that a combination of virus and drug effectively impair overall viability of pancreatic tumour organoids from a clinically relevant mouse model of pancreatic cancer.

### Ad5_NULL_A20.FCU1 in combination with 5-FC reduces viability in patient derived PDAC organoids

Previous findings have reported on the clinical relevance of patient derived organoids (PDOs) from PDAC samples [29, 30]. We sought to investigate whether pancreatic tumour patient-derived organoids could be transduced with an αvβ6 integrin selective virus incorporating CD and UPRTase, and sensitised to 5-FC treatment.

**Fig. 5:**
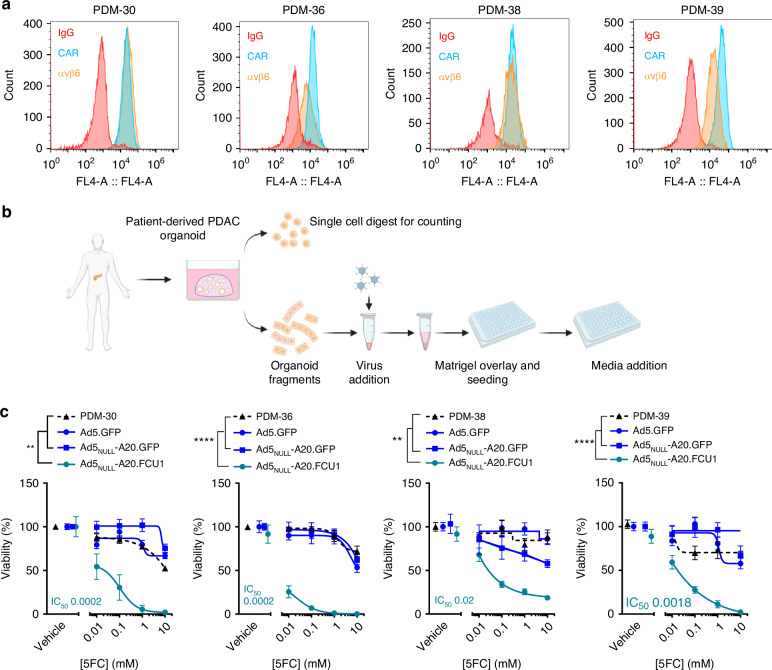
Patient-derived PDAC organoids show variable sensitivities to Ad5_NULL_-A20.FCU1 and 5-FC combination treatment. **a** αvβ6 Integrin and CAR screening of patient-derived PDAC organoids (PDM-30, PDM36, PDM-38,PDM-39) by flow cytometry. **b** Schematic of organoid generation and transduction with Ad5_NULL_-A20.FCU1 or Ad5.GFP. **c** Ad5_NULL_-A20.FCU1 display heterogeneity in capacity to sensitise patient-derived pancreatic tumour organoids to 5-FC treatment. PDM-30, PDM-36, PDM-38 and PDM-39 organoids were transduced as fragments with 5000 vp/cell of virus for 24 h prior to treatment with a range of 5-FC doses. Cell viability was estimated by CellTiter Glo®. Data following 5–6 days in culture are represented as mean ± SEM, *n* = 3. Two-way ANOVA with Tukey’s post hoc test was used.

PDAC-derived organoids were derived previously as part of the Human Cancer Models Initiative and cultured as described [31], with tumour-status of organoids confirmed by DNA sequencing. To assess the potential of Ad5_NULL_-A20 and Ad5 vectors to transduce PDAC PDOs, we analysed the expression levels of αvβ6 integrin and CAR by flow cytometry on single cell digests of organoid structures (Fig. 5a). All organoid lines tested were positive for αvβ6 integrin and CAR expression. This is in corroboration with previous findings in the literature which report >90% PDAC patient histology samples demonstrate high αvβ6 integrin expression [16].

Using protocols optimised in KPC-derived mouse organoids, we transduced organoid fragments with Ad5.GFP, Ad5_NULL_-A20.GFP or Ad5_NULL_-A20.FCU1 for 24 h prior to treatment with or without the prodrug 5-FC (Fig. 5b). We evaluated the effects of Ad5.GFP, Ad5_NULL_-A20.FCU1 and 5-FC in combination or alone in PDAC organoids derived from four different patients. Phase-contrast and corresponding fluorescence images of HCM-CSHL-0079-C25 (ATCC® PDM30™); PDM-30, HCM-CSHL-0089-C25 (ATCC® PDM36™); PDM-36, HCM-CSHL-0091-C25 (ATCC® PDM38™); PDM-38 and HCM-CSHL-0092-C25 (ATCC® PDM39™); PDM-39 show that organoids were successfully transduced with Ad5.GFP and Ad5_NULL_-A20.GFP using this method (Supplementary Fig. S5). We found that organoids alone or transduced by Ad5.GFP and Ad5_NULL_-A20.GFP control vectors and treated with 0 mM and 10 mM 5-FC exhibited few differences in overall viability as measured by a viability assay readout (Fig. 5c). Organoid viability was reduced when treated with Ad5_NULL_-A20.FCU1 in combination with 5-FC, resulting in IC_50_ values ranging from 0.0002 mM to 0.02 mM across the various models (Fig. 5c). Organoids that expressed the highest levels of αvβ6 integrin by flow cytometry were found to be sensitive to treatment, such as PDM-30; however, no clear correlation could be found between sensitivity and degree of αvβ6 integrin expression levels. Given that PDM-38 showed the least sensitivity to FCU1 expression and 5-FC combination (Fig. 5c) of all the models tested, this suggests that the presence of a subset αvβ6 integrin is sufficient to cause toxicity via a bystander effect, corroborating previous findings in the literature [5]. These results further demonstrate promising evidence of the feasibility of using tumour organoids in pre-clinical testing of replication-deficient virotherapies.

### Ad5_NULL_-A20.FCU1 in combination with 5-FC limits tumour growth in a αvβ6 integrin-positive xenograft model

**Fig. 6:**
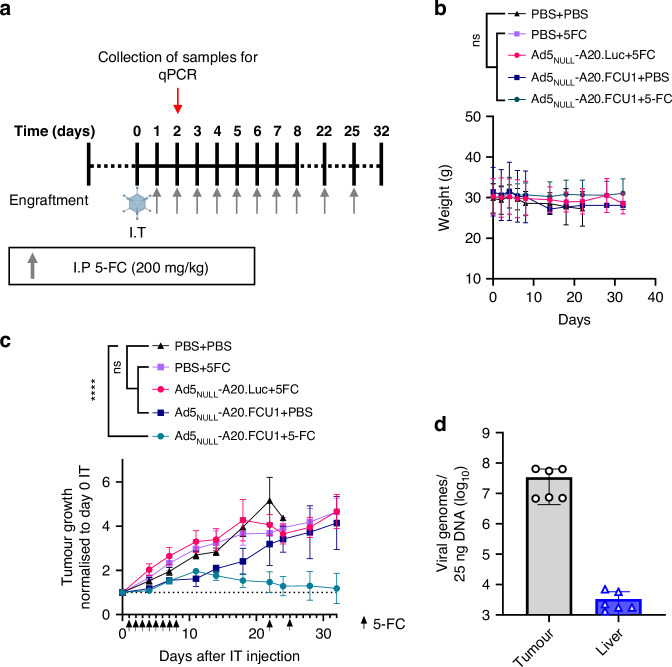
Ad5_NULL_-A20.FCU1 sensitises a CFPAC1 xenograft model to 5-FC treatment. **a** Schematic of in vivo experiment and treatment schedule following engraftment with a CFPAC1 cell line. **b** Weight changes of mice measurement throughout treatment. Data shown as mean ± S.D from a minimum of *n* = 4 mice per treatment condition (Tukey’s multiple comparison test). **c** CFPAC1 (αvβ6^high^) xenograft model was generated by subcutaneous implantation of cells bilaterally in flanks of nude mice. Following tumour formation mice were injected intratumorally (I.T) with 3 × 10^10^ vp of Ad5_NULL_-A20.FCU1, Ad5_NULL_-A20.Luc (Ad5_NULL_-A20 containing a luciferase transgene control), or PBS. 5-FC (200 mg/kg/d) or PBS vehicle control was delivered intraperitoneally (I.P) daily for 8 days, then again on days 22 and days 25. Tumour volume is presented as relative to size measured on Day 0 of IT injections. Data are represented as mean ± S.D from a minimum of *n* = 5 tumours per treatment group (Two way ANOVA with Tukey’s multiple comparison test). **d** Viral genome copy number in organs at 48 h following IT delivery of Ad5_NULL_-A20.FCU1. Adenovirus genome copy number was established from tissues excised from CFPAC1 xenografts, and determined by qPCR for the Ad hexon gene. Data presented as mean ± S.D from *n* = 2 mice in technical replicates.

To evaluate the overall efficacy of our candidate FCU1 virotherapy in vivo, we performed bilateral subcutaneous xenografts with CFPAC1 (αvβ6^high^) cells in both flanks of nude mice. When tumours reached 5 mm they were injected with 3 × 10^10^ vp of Ad5_NULL_-A20.FCU1, Ad5_NULL_-A20.Luciferase as a transgene control, or PBS. 24 h post I.T delivery of Ads, vehicle control (PBS) or 5-FC (200 mg/kg) were administered daily for 8 days by I.P, then again at days 22 and 25 (Fig. 6a). To ensure no toxicity was observed as a result of Ad treatment, animals were weighed every 1–3 days throughout the experiment duration. No significant weight fluctuations occurred in any of the treatment groups (Fig. 6b). We found that Ad5_NULL_-A20.FCU1 in combination with 5-FC resulted in significantly reduced tumour volumes compared to mice treated with I.T PBS and I.P PBS (*p* < ****, Fig. 6c). Tumours administered with Ad5_NULL_-A20.FCU1 and 5-FC showed a significant difference in growth compared to those treated with PBS, suggesting that the FCU1 transgene and 5-FC in combination were able to selectively inhibit tumour growth. No change in overall tumour growth was observed in a transgene control Ad5_NULL_-A20.Luc +/− 5-FC compared to I.T PBS + I.P PBS. Quantitation of viral load by qPCR from mouse tissues harvested at 48 h post IT administration of Ad5_NULL_-A20.FCU1 showed that the virotherapy showed increase accumulation in the tumour compared to the liver (Fig. 6d). Taken together, these findings corroborate observations in vitro and ex vivo whereby Ad5_NULL_-A20.FCU1 sensitise αvβ6 integrin positive PDAC cells to treatment with 5-FC, a drug that is routinely used as a non-toxic anti-fungal agent, to inhibit tumour growth in vivo.

## Discussion

Treatment strategies for PDAC rely heavily on surgical resection and chemotherapeutic delivery in the adjuvant or neoadjuvant setting. Systemic delivery of 5-FU is not only associated with severe side effects but lacks full therapeutic efficacy due to limited drug delivery and the rapid development of drug resistance [18].

In this study, we generated Ad5 and Ad5_NULL_-A20 vectors armed with FCY1 and FCU1 transgenes and evaluated their overall effect on viability in the presence and absence of 5-FC, in a panel of pancreatic cell lines with varying known levels of CAR and αvβ6 integrin. This strategy is designed to overcome the systemic toxicity of 5-FU as only transduced cells will activate the prodrug selectively in αvβ6 expressing cancer cells. We found that the Ad5 mediated transfer of FCY1 in tumour cells can successfully sensitize CAR+ cells to 5-FC treatment and cause growth arrest in vitro. We found the bifunctional protein (FCU1) that combines the two enzymatic activities of CD and UPRTase, increased in vitro sensitivity to 5-FC compared to Ad5.FCY1-transduced cells, corroborating previous findings in the literature [5, 26]. The efflux of nucleoside analogues is a key mechanism that enables treatment resistance to 5-FU [18]. The direct delivery of 5-FUMP, an active metabolite of 5-FU, by UPRTase-dependent conversion of 5-FC could therefore enhance toxicity as well as overcome resistance to 5-FU treatment. Replication-deficient Ad5_NULL_-A20 expressing FCY1/FCU1 was able to successfully transduce and sensitise αvβ6+ pancreatic cells to 5-FC treatment. PT45 cells (CAR+/αvβ6−) were only sensitised to 5-FC treatment following transduction with Ad5.FCY1/FCU1 suggesting that Ad5_NULL_-A20.FCY1/FCU1 is selective only to αvβ6+ cells. Importantly we show this novel therapeutic approach is also able to dramatically inhibit the growth of αvβ6 positive PDAC cells in patient derived ex vivo tumour organoids and in vivo.

Enzyme-prodrug systems for chemotherapeutic delivery to tumours via viral vectors have previously been described, with studies demonstrating localised delivery of 5-FU via CDase/UPRTase conversion of 5-FC in vitro and in xenograft models. This has been reported using replication-deficient and oncolytic adenoviruses [5, 6, 26], vaccinia virus [32], cowpox virus [33] and measles vaccine virus (MeV) [34] genetically engineered to express CDase/UPRTase enzymes. Whilst studies have shown the improved overall safety profile of chemotherapeutic delivery, significant challenges have been identified in the selective transduction of tumour cells that can effectively induce an anti-tumour effect. We have previously shown that Ad5_NULL_-A20 is a highly tumour-selective platform, capable of selectively transducing tumour cells with αvβ6 integrin [14, 15]. The epithelial integrin αvβ6 is expressed at minimal levels in normal healthy adult tissue with previous studies identifying weak localised expression on epithelial cells of the airway, colon and placenta [35, 36] and commonly upregulated during epithelial cell remodelling including tissue repair [37] and tumourigenesis [38]. Aberrant expression of αvβ6 has been reported in numerous solid cancers and metastases [35, 38], including PDAC, whereby αvβ6 integrin is highly prevalent in almost 90% of patient tumours [16] and therefore represents a promising target for selective therapeutic delivery.

We further evaluated the efficacy of Ad5_NULL_-A20.FCU1 in combination with 5-FC in 3D organoids from clinically-relevant sources. Organoids derived from patient tumours or KPC mouse models effectively recapitulate multiple features associated with PDAC such as tumour heterogeneity [39], cell signalling and overall tissue architecture [29, 40–42]. Patient derived PDAC models successfully mirror a complex tumour landscape and are therefore highly predictive models of therapeutic efficacy both in vivo [43] and in paired primary tumours [40]. Pre-clinical studies evaluating oncolytic virus efficacy have largely relied on 2D cell lines, which fail to mimic PDAC tumour physiology, limiting their ability to fully recapitulate virus binding and internalisation within a tumour [44]. Here, we optimised transduction for delivery of replication-deficient viruses that could successfully transduce organoids with virus doses comparable to those used in vitro.

We found that KPC-derived organoids retained similar αvβ6 integrin expression levels to corresponding in vivo tissues, and were amenable to Ad-based transduction as visualised by GFP-positive organoids. We further used patient derived pancreatic ductal adenocarcinoma organoids. The addition of 5-FC to the Ad5_NULL_-A20.FCU1 -transduced organoids led to a significant decrease in organoid viability and growth in comparison to control conditions. PDAC organoids from different patients displayed varying levels of sensitivity that could not fully be correlated to expression of αvβ6, similar to previous findings investigating oncolytic viral entry in organoids whereby membrane integrins alone did not fully predict viral entry capacity [43]. Given the complexity of viral cell entry and trafficking to the nucleus, it is possible this would vary across different patients. However, for the VDEPT described in this study, the accumulation of 5-FU and 5-FUMP from transduced cells could be sufficient to cause toxicity to the local tumour cell population, bypassing the need for viral cell entry in all tumour cells.

This work now makes additional in vivo studies an attractive future research avenue to overcome the limitations of using subcutaneous engraftment to model PDAC [45], and help build a body of evidence to ascertain whether a VDEPT strategy utilising a Ad5_NULL_-A20 tumour-selective vector would show effective anti-tumour activity following Intravenous (I.V) delivery, in combination with 5-FC. Although I.T delivery of our candidate virus shows a promising response in vivo in combination with I.P delivery of 5-FC, systemic delivery of would be a necessary strategy in the clinic, particularly for the treatment of tumours that are typically non-accessible, such as PDAC and metastatic disease. Given that 5-FC is a non-toxic prodrug routinely administered as an antifungal in the clinic and can be tolerated at high doses, systemic delivery of the pro-drug would give limited off-target toxicity. An important component of this work would further establish whether high anti-Ad5 neutralising antibodies would impact the efficacy and potential therapeutic window in this precision VDEPT approach. Adopting this VDEPT strategy in oncolytic Ad5_NULL_-A20 could further enhance tumour cell killing, as demonstrated previously in Ad5/3 [5, 7]. A current limitation of testing viral transduction in organoids and the CFPAC1 in vivo models described in this study is their lack of immune-cell subsets. Co-culture assays would therefore be essential future research avenue to determine whether novel viruses have the capacity to stimulate an immune response.

In summary, this study demonstrated that the modification of the tumour selective Ad5_NULL_-A20 platform to incorporate suicide gene therapy resulted in effective tumour cell killing in vitro and ex vivo and was effective when delivered with relatively low doses of 5-FC prodrug. Our data however provide a strong rationale for clinical translation of Ad5_NULL_-A20.FCU1 as a viral vector to combine with 5-FC treat αvβ6 integrin expressing pancreatic cancer cells. This platform could also be effective in other αvβ6 expressing cancer types such as gastric and ovarian cancer [46, 47]. We conclude that Ad5_NULL_-A20.FCU1 is a promising candidate for clinical development for localised in-tumour chemotherapeutic delivery.

## Supplementary information

Supplementary Figures S1 - S5

## Data Availability

All data within this manuscript is available from the authors upon reasonable request.
