# Supplementary material for: The αvβ6 integrin specific virotherapy, Ad5NULL-A20.FCU1, selectively delivers potent “in-tumour” chemotherapy to pancreatic ductal adenocarcinoma
Source: Br J Cancer. 2024 Oct 5;131(10):1694–706. doi: 10.1038/s41416-024-02869-3 (PMC11555051; doi:10.1038/s41416-024-02869-3)
Supplement: Supplementary file 1 — Supplementary Figures S1 - S5 [file 41416_2024_2869_MOESM1_ESM.pptx]

## Slide 1
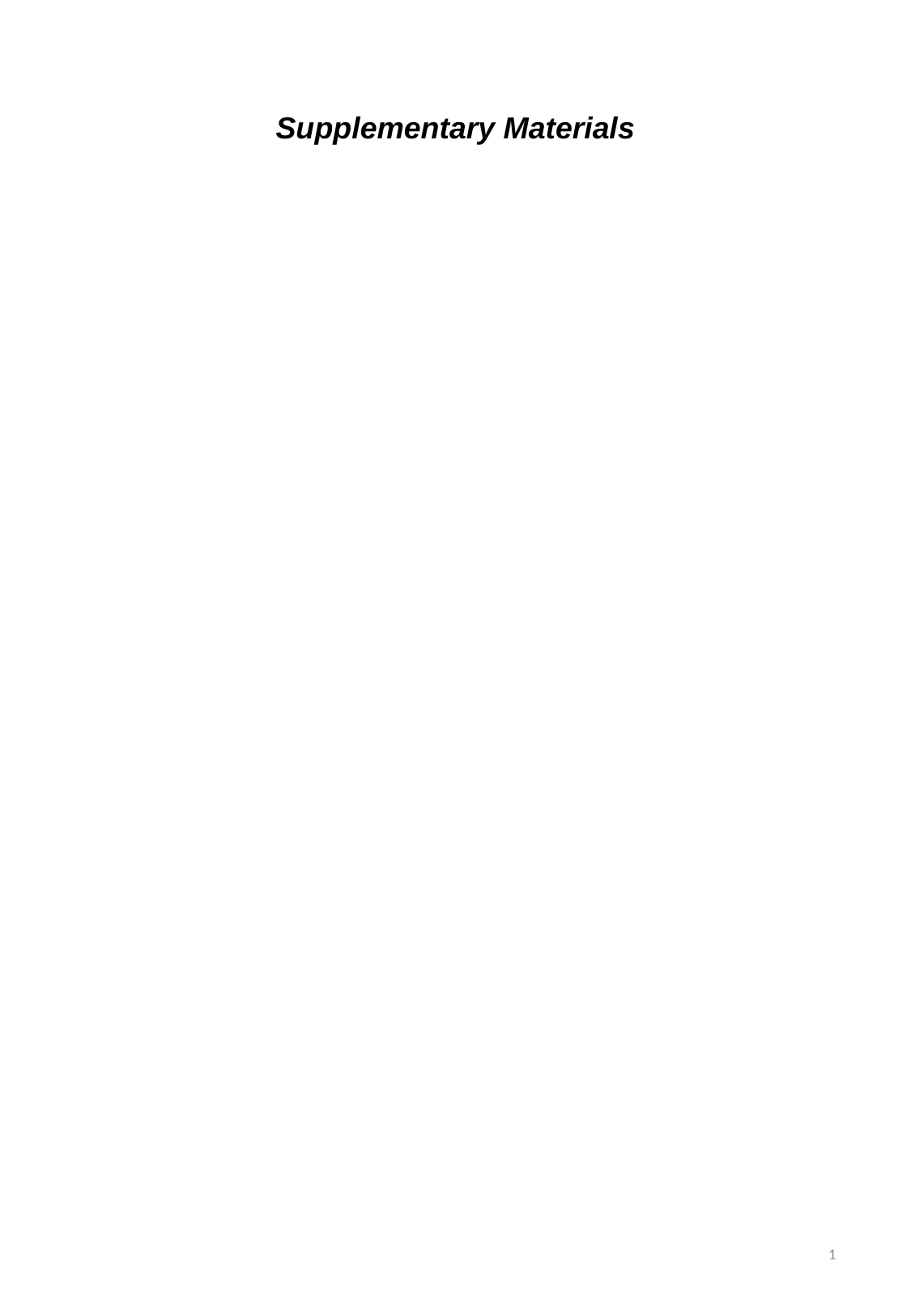

Supplementary Materials
1

## Slide 2
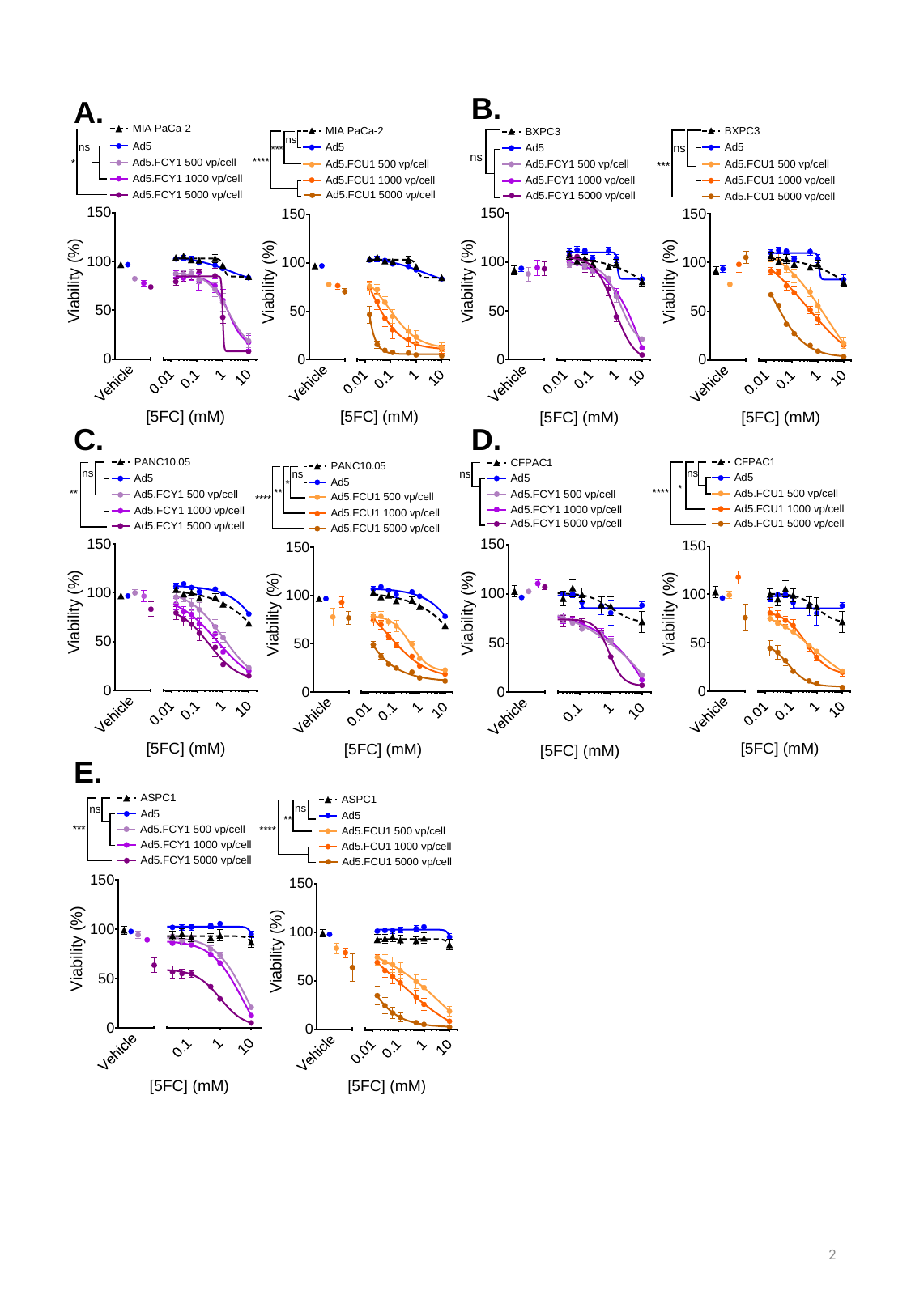

B.
A.
C.
D.
E.
2

## Slide 3
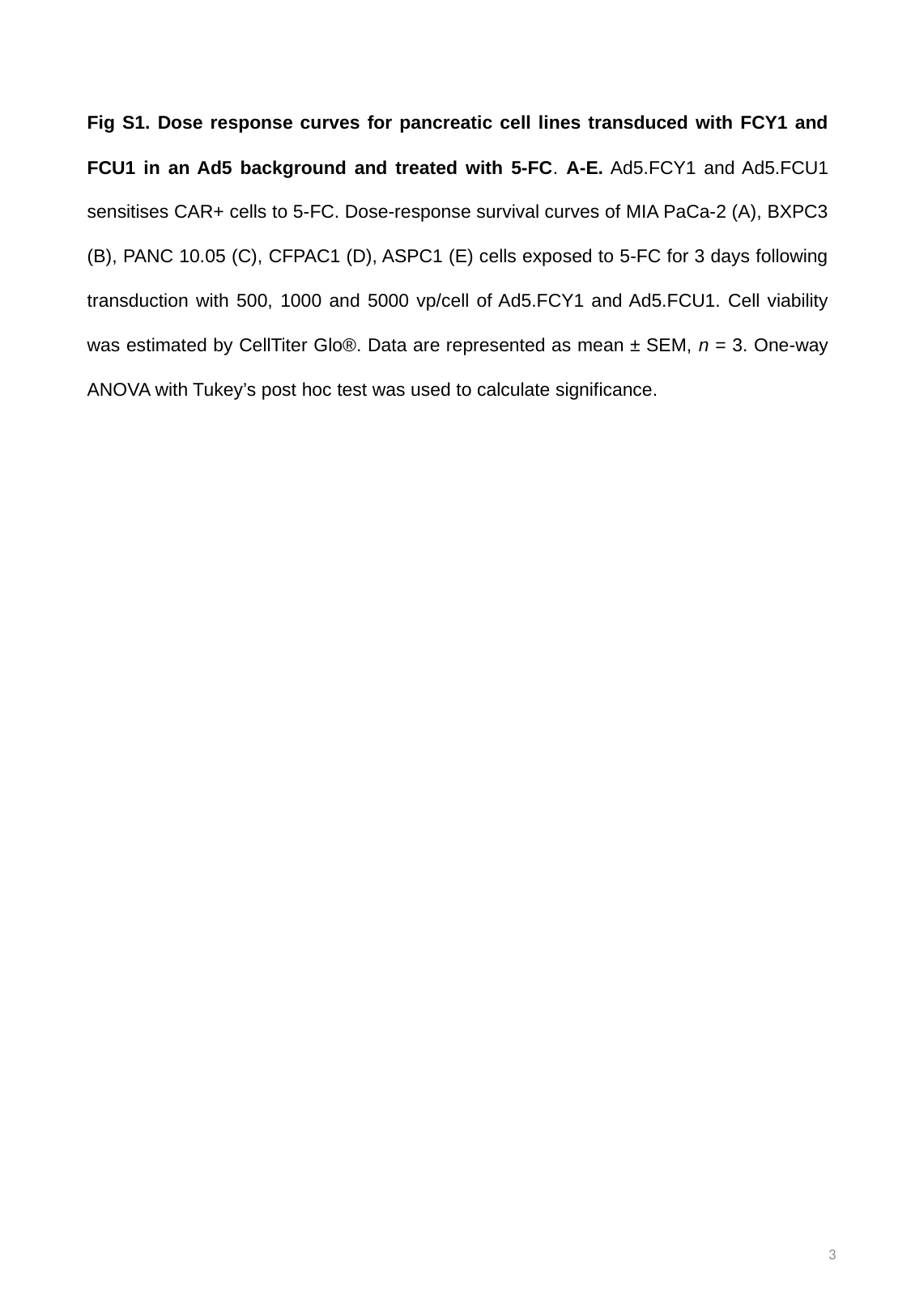

Fig S1. Dose response curves for pancreatic cell lines transduced with FCY1 and FCU1 in an Ad5 background and treated with 5-FC. A-E. Ad5.FCY1 and Ad5.FCU1 sensitises CAR+ cells to 5-FC. Dose-response survival curves of MIA PaCa-2 (A), BXPC3 (B), PANC 10.05 (C), CFPAC1 (D), ASPC1 (E) cells exposed to 5-FC for 3 days following transduction with 500, 1000 and 5000 vp/cell of Ad5.FCY1 and Ad5.FCU1. Cell viability was estimated by CellTiter Glo®. Data are represented as mean ± SEM, n = 3. One-way ANOVA with Tukey’s post hoc test was used to calculate significance.
3

## Slide 4
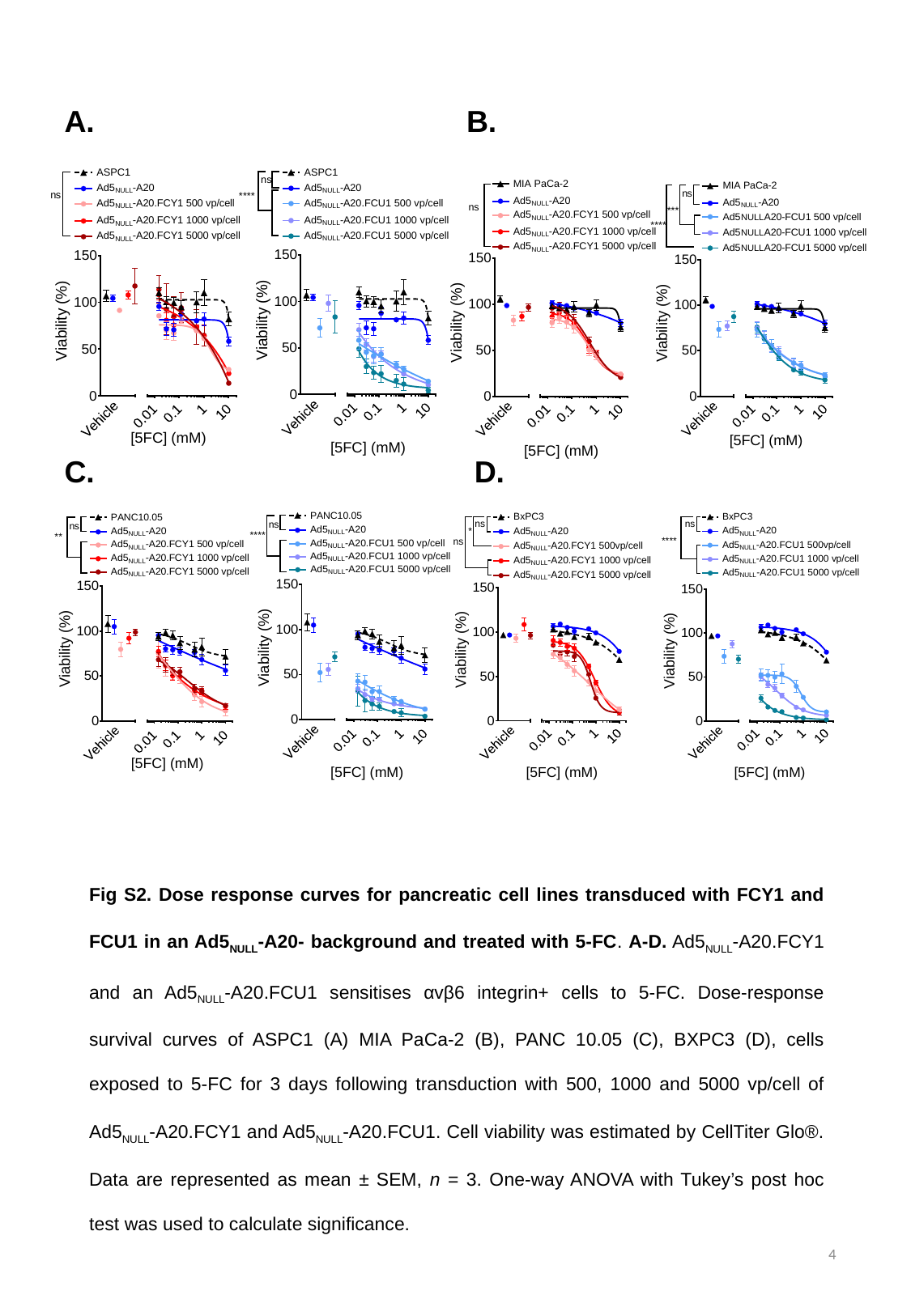

A.
B.
C.
D.
Fig S2. Dose response curves for pancreatic cell lines transduced with FCY1 and FCU1 in an Ad5NULL-A20- background and treated with 5-FC. A-D. Ad5NULL-A20.FCY1 and an Ad5NULL-A20.FCU1 sensitises αvβ6 integrin+ cells to 5-FC. Dose-response survival curves of ASPC1 (A) MIA PaCa-2 (B), PANC 10.05 (C), BXPC3 (D), cells exposed to 5-FC for 3 days following transduction with 500, 1000 and 5000 vp/cell of Ad5NULL-A20.FCY1 and Ad5NULL-A20.FCU1. Cell viability was estimated by CellTiter Glo®. Data are represented as mean ± SEM, n = 3. One-way ANOVA with Tukey’s post hoc test was used to calculate significance.
4

## Slide 5
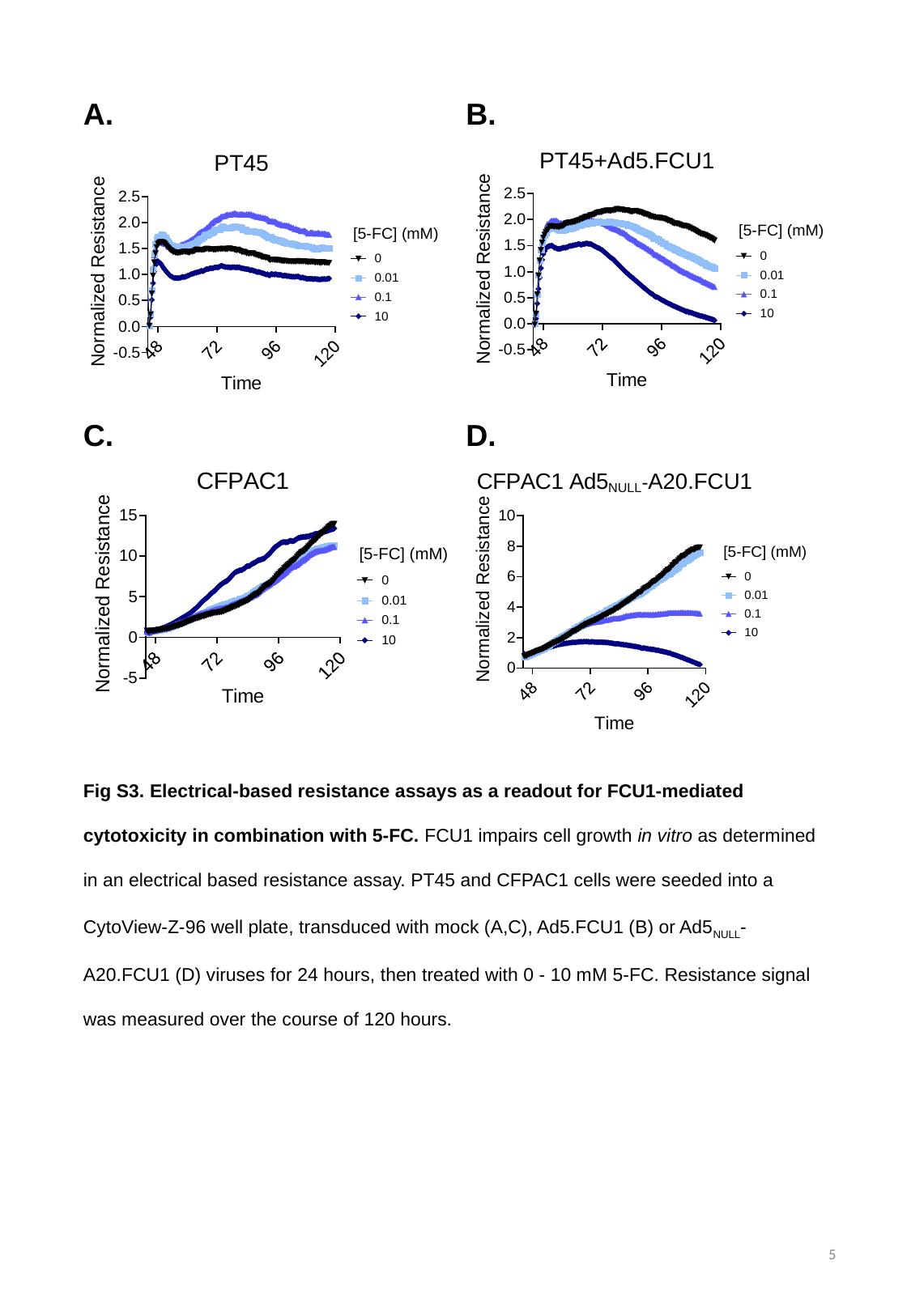

A.
B.
C.
D.
Fig S3. Electrical-based resistance assays as a readout for FCU1-mediated cytotoxicity in combination with 5-FC. FCU1 impairs cell growth in vitro as determined in an electrical based resistance assay. PT45 and CFPAC1 cells were seeded into a CytoView-Z-96 well plate, transduced with mock (A,C), Ad5.FCU1 (B) or Ad5NULL-A20.FCU1 (D) viruses for 24 hours, then treated with 0 - 10 mM 5-FC. Resistance signal was measured over the course of 120 hours.
5

## Slide 6
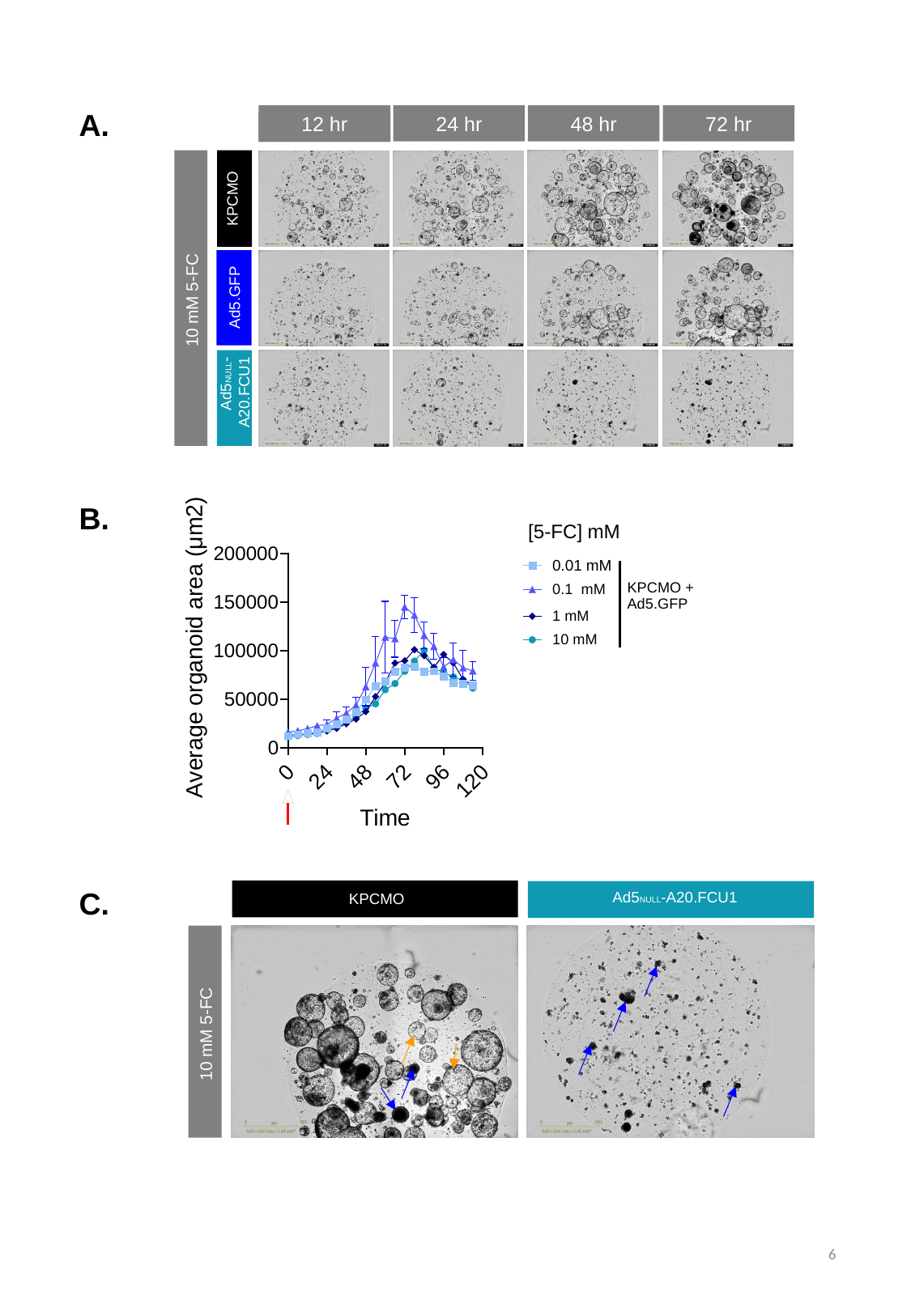

A.
12 hr
24 hr
48 hr
72 hr
KPCMO
10 mM 5-FC
Ad5.GFP
Ad5NULL-A20.FCU1
B.
C.
KPCMO
Ad5NULL-A20.FCU1
10 mM 5-FC
6

## Slide 7
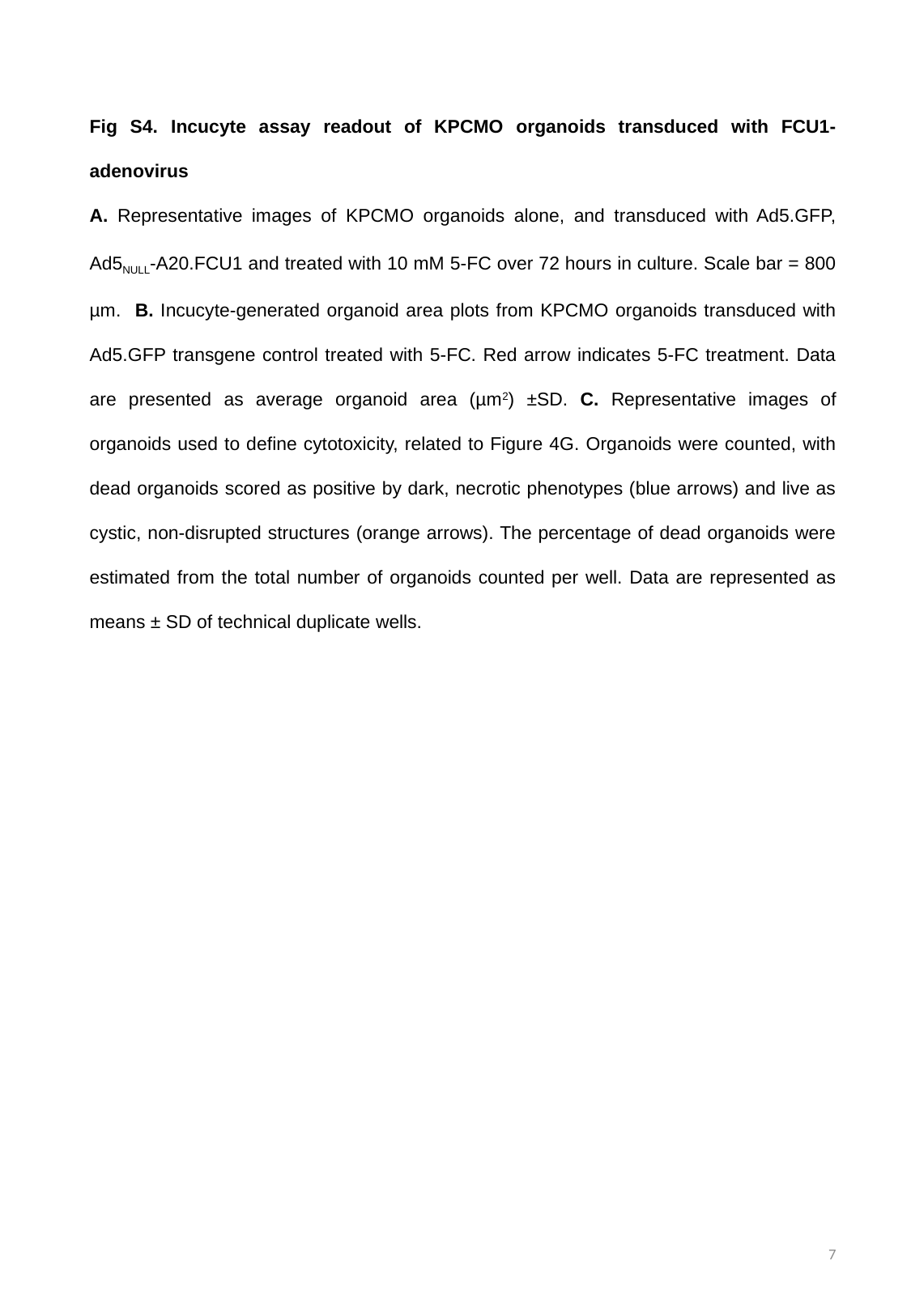

Fig S4. Incucyte assay readout of KPCMO organoids transduced with FCU1-adenovirus
A. Representative images of KPCMO organoids alone, and transduced with Ad5.GFP, Ad5NULL-A20.FCU1 and treated with 10 mM 5-FC over 72 hours in culture. Scale bar = 800 µm. B. Incucyte-generated organoid area plots from KPCMO organoids transduced with Ad5.GFP transgene control treated with 5-FC. Red arrow indicates 5-FC treatment. Data are presented as average organoid area (µm2) ±SD. C. Representative images of organoids used to define cytotoxicity, related to Figure 4G. Organoids were counted, with dead organoids scored as positive by dark, necrotic phenotypes (blue arrows) and live as cystic, non-disrupted structures (orange arrows). The percentage of dead organoids were estimated from the total number of organoids counted per well. Data are represented as means ± SD of technical duplicate wells.
7

## Slide 8
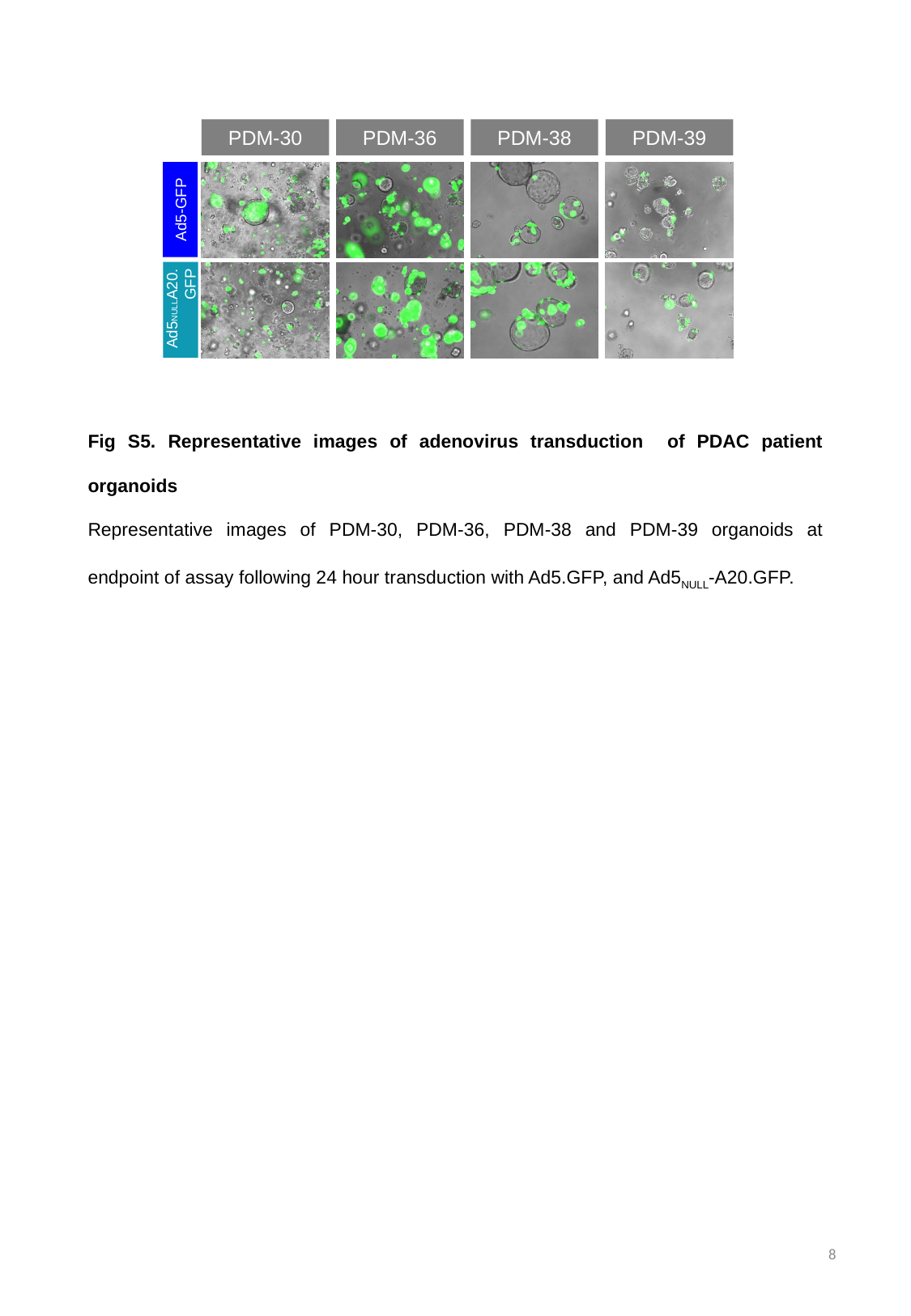

PDM-30
PDM-36
PDM-38
PDM-39
Ad5-GFP
Ad5NULLA20.GFP
Fig S5. Representative images of adenovirus transduction of PDAC patient organoids
Representative images of PDM-30, PDM-36, PDM-38 and PDM-39 organoids at endpoint of assay following 24 hour transduction with Ad5.GFP, and Ad5NULL-A20.GFP.
8
